# Predicting the Next-Day Perceived and Physiological Stress of Pregnant Women by Using Machine Learning and Explainability: Algorithm Development and Validation

**DOI:** 10.2196/33850

**Published:** 2022-08-02

**Authors:** Ada Ng, Boyang Wei, Jayalakshmi Jain, Erin A Ward, S Darius Tandon, Judith T Moskowitz, Sheila Krogh-Jespersen, Lauren S Wakschlag, Nabil Alshurafa

**Affiliations:** 1 McCormick School of Engineering Northwestern University Evanston, IL United States; 2 Northwestern University Feinberg School of Medicine Chicago, IL United States

**Keywords:** explainability, just-in-time interventions, machine learning, prenatal stress, stress prediction, wearable, mobile phone

## Abstract

**Background:**

Cognitive behavioral therapy–based interventions are effective in reducing prenatal stress, which can have severe adverse health effects on mothers and newborns if unaddressed. Predicting next-day physiological or perceived stress can help to inform and enable pre-emptive interventions for a likely physiologically and perceptibly stressful day. Machine learning models are useful tools that can be developed to predict next-day physiological and perceived stress by using data collected from the previous day. Such models can improve our understanding of the specific factors that predict physiological and perceived stress and allow researchers to develop systems that collect selected features for assessment in clinical trials to minimize the burden of data collection.

**Objective:**

The aim of this study was to build and evaluate a machine-learned model that predicts next-day physiological and perceived stress by using sensor-based, ecological momentary assessment (EMA)–based, and intervention-based features and to explain the prediction results.

**Methods:**

We enrolled pregnant women into a prospective proof-of-concept study and collected electrocardiography, EMA, and cognitive behavioral therapy intervention data over 12 weeks. We used the data to train and evaluate 6 machine learning models to predict next-day physiological and perceived stress. After selecting the best performing model, Shapley Additive Explanations were used to identify the feature importance and explainability of each feature.

**Results:**

A total of 16 pregnant women enrolled in the study. Overall, 4157.18 hours of data were collected, and participants answered 2838 EMAs. After applying feature selection, 8 and 10 features were found to positively predict next-day physiological and perceived stress, respectively. A random forest classifier performed the best in predicting next-day physiological stress (F1 score of 0.84) and next-day perceived stress (F1 score of 0.74) by using all features. Although any subset of sensor-based, EMA-based, or intervention-based features could reliably predict next-day physiological stress, EMA-based features were necessary to predict next-day perceived stress. The analysis of explainability metrics showed that the prolonged duration of physiological stress was highly predictive of next-day physiological stress and that physiological stress and perceived stress were temporally divergent.

**Conclusions:**

In this study, we were able to build interpretable machine learning models to predict next-day physiological and perceived stress, and we identified unique features that were highly predictive of next-day stress that can help to reduce the burden of data collection.

## Introduction

### Background

Welcoming a new member to the family is cause for celebration but can also lead to substantial stress, particularly for mothers. A systematic review of perinatal depression (PD) predictors identified prenatal stressors as either episodic (eg, life events or daily hassles) or chronic (eg, parenting stress, perceived stress, and chronic strain) [1]. Lack of social support, stressful life events, domestic violence, low socioeconomic status, and past history of depression contribute significantly to increased prenatal stress [2,3]. Maternal stress can lead to preterm birth or low birth weight [4,5], which are leading causes of infant mortality in the United States [6,7], or structural malformations [8] and psychosocial impairment [9].

To mitigate these negative outcomes, a number of interventions have been developed and tested to reduce stress in pregnant women, often using the principles of mindfulness [10-15] and cognitive behavioral therapy (CBT) [16-19] in group or individual format. A key characteristic of many CBT-based interventions is the inclusion of personal practice or homework between intervention sessions to facilitate adoption of newly learned skills [20]. Such homework can take the form of technologically supported just-in-time (JIT) interventions [21], which in the case of maternal stress can enhance effectiveness of stress-reducing techniques. Incorporation of JIT interventions is facilitated by technology that participants can receive on mobile phones. Use of JIT interventions is associated with improvement in mental health symptoms and conditions that these interventions target [22-24]. However, the timing of interventions may affect participation [25], especially given that JIT interventions typically require individuals to perform an action in the moment to achieve desired outcomes. To appropriately target stress with JIT interventions, it is necessary to identify factors that are most predictive of stress to develop a mechanism to proactively detect and deliver a timely preventive intervention.

However, there is no singular definition of stress and the mechanisms underlying physiological and perceived stress are different, requiring different means of detection and prediction [26]. Physiological stress that persists from one day to the next, or *residual stress*, can be most damaging to neurovascular health and lead to chronic diseases [27,28]. Although it is unclear how perceived stress maps onto future disease state, perceived stress can be debilitating and linked to poor life satisfaction [29]. The ability to predict next-day stress, whether physiological or perceived, and to understand predictors of either type of stress may allow for advanced scheduling of JIT interventions that help to reduce or prevent next-day stress.

Machine learning models have been used to successfully predict both physiological and perceived stress; however, few models predict beyond the near future while also explaining the driving forces behind the predictions. Several sensing systems have been designed to forecast physiological stress in the future [30,31]. However, studies predicting physiological stress are often performed in a laboratory owing to the limited feasibility of frequent stress assessments in the wild [32]. Other studies have captured both perceived and physiological stress but only consider physiological stress when determining ground truth for the machine-learned models [33]. The few examples of next-day stress prediction using prior days’ data [34,35] either focused on testing the difference between generalized and personalized models or focused primarily on prediction of perceived stress.

Being able to predict stress earlier and with minimal data collection burden while assessing the interpretability of the model will allow researchers to improve their understanding of the learned model, increasing their understanding of how the model determines stress the next day and informing the design of JIT intervention. Models can use global explanations, which attempt to describe the overall functionality of the learned model (eg, feature importance), or local explanations, which are aimed at explaining the model’s reasoning for a specific instance. Some types of explanations such as Shapley Additive Explanations (SHAP) [36] enable greater interpretability of models and are considered model agnostic, providing both global and local explanations. SHAP can be used to create global explanations by aggregating SHAP values to create feature importance, summary, and dependence plots. SHAP values are feature attributions that act as driving forces either contributing to the prediction or not. Ultimately, these results can inform means of low-burden early stress detection, which in turn enables scheduling of JIT intervention content that prevents future stress and its correlates.

### Objectives

In this pilot study, we aimed to predict next-day stress in pregnant women who participated in a perinatal stress reduction course. Specifically, we obtained data from sensors and participant self-report and then used several machine learning models to find the best performer. We evaluated the potential of our model to predict next-day stress and applied an explainability model to provide meaning to our predictions.

## Methods

### Study Design

We collaborated with a private university’s obstetrics and gynecology clinic to recruit pregnant women into our study. To be eligible for enrollment, women had to be aged ≥18 years, enrolled at 10 to 18 weeks’ gestation with a singleton pregnancy, and own a smartphone. Women were excluded if they had a known medical or pregnancy complication that may place their infant at risk for neurological disorders or significant mental health disorders.

Upon enrollment, participants received a 12-week person-to-person intervention called the *Mothers and Babies* (MB) course [19] from a master’s-level social worker and wore a mobile electrocardiography (ECG) sensor, BioStampRC (MC10), to capture heart rate (HR) data (Figure S1 in Multimedia Appendix 1). Throughout the study, participants received SMS text messages on their mobile phones in the form of ecological momentary assessment (EMA) surveys for self-reported stress assessment. During the first intervention session, participants were shown the BioStampRC sensor and trained to use it. Subsequent MB intervention sessions (1:1 interventions) were delivered every 1 to 2 weeks by the same social worker, either in person or through the phone. At the end of 12 intervention sessions, participants returned the sensor and were asked to provide feedback on the usability and wearability of the sensor, as well as the acceptability of the EMA surveys through a semistructured exit interview. The women received US $200 compensation for completing the study.

**Figure 1:**
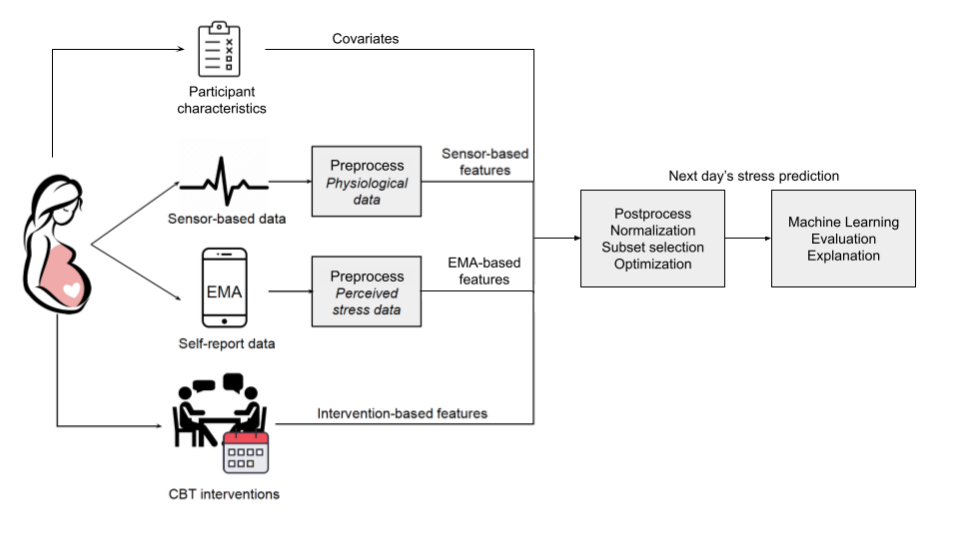
Data collection and processing pipeline for predicting next-day stress. CBT: cognitive behavioral therapy; EMA: ecological momentary assessment.

**Figure 2:**
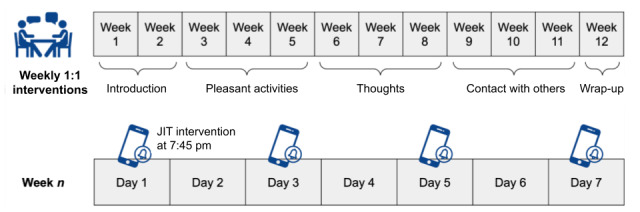
Mothers and Babies program intervention schedule and content. JIT: just-in-time.

After the completion of the study, we performed data extraction and preprocessing and then applied machine learning models and a SHAP explainability model to identify predictors of next-day physiological and perceived stress. Overviews of the study design and MB program are shown in Figures 1 and 2, respectively.

### Ethics Approval

The study was approved by Northwestern University’s institutional review board (approval number: STU00205776), and all women provided written informed consent before enrollment.

### MB Course

The MB course is an effective, evidence-based intervention originally developed for preventing postpartum depression [37]. The MB course comprises a dozen 1:1 sessions, each designed to last for 15 to 20 minutes. The intervention provides a toolkit of cognitive behavior approaches to promote increasing healthy behaviors, helpful thoughts, and social support within the context of parenting and bonding with one’s baby. Throughout the course, a variety of mindfulness practices are introduced along with mindfulness tips to support integration into daily life. The first 2 MB sessions introduce the cognitive behavior model and discuss the relationship between one’s mood and stress and one’s behaviors, thoughts, and social interactions. The pleasant activities module (sessions 3-5) focuses on identifying and increasing engagement in pleasant activities alone, with others, and with one’s baby. The thoughts module (sessions 6-8) focuses on strategies to increase helpful thoughts and decrease unhelpful thoughts. The contact with others module (sessions 9-11) focuses on increasing positive and supportive interactions with other people.

The MB sessions were delivered weekly or every other week, occasionally with 2 sessions delivered in 1 visit to facilitate timely completion of the intervention, allowing for participant scheduling needs. In addition, participants received a JIT intervention, consisting of 4 SMS text messages, every other day at 7:45 PM on their mobile phones after each completed 1:1 intervention. The SMS text messages included brief messages and links to external content (eg, worksheets, videos, and guided mindfulness practices) and were designed to reinforce the most recent 1:1 intervention content and to encourage skill practice so that the skills become more frequently used to manage stress in one’s daily life [38].

### Assessments

Baseline demographics and pregnancy history were collected. Depression symptoms at baseline were measured using the Edinburgh Postnatal Depression Scale (EPDS), a validated 10-item self-report assessment that is the most frequently used PD screening tool. The EPDS assesses symptoms of anxiety and depression, both of which are frequent features in perinatal mood disorders and excludes symptoms that are commonly experienced during pregnancy and the postpartum period, such as changes in sleep and appetite. Individual responses are scored on a scale from 0 to 3, with 0 indicating no symptoms of depression and 3 indicating high frequency of depression symptoms. The total score ranges from 0 to 30, with higher scores indicating increased severity and frequency of depression symptoms.

We collected ECG data using a patch-like flexible sensor, BioStampRC, that was placed on the left side of the participant’s chest. The BioStampRC is effective in using HR-based features to predict physiological and perceived stress in pregnant women [39]. Participants used a study-provided tablet to start and stop sensor recording and to upload completed recordings to a secure cloud platform at the end of the day. Participants were asked to wear the device during waking hours throughout the 12-week study and could take a few days off to prevent adverse effects of wearing a strong adhesive in the same location every day.

At the time of study enrollment, all participants were asked to identify their usual daily wake and sleep times, and daily EMA questionnaires were programmed to be sent 5 times a day at evenly distributed intervals within each participant’s waking hours. Each EMA questionnaire consisted of 12 questions (Table S1 in Multimedia Appendix 1).

### Model Development

#### Sensor Data Processing

**Figure 3:**
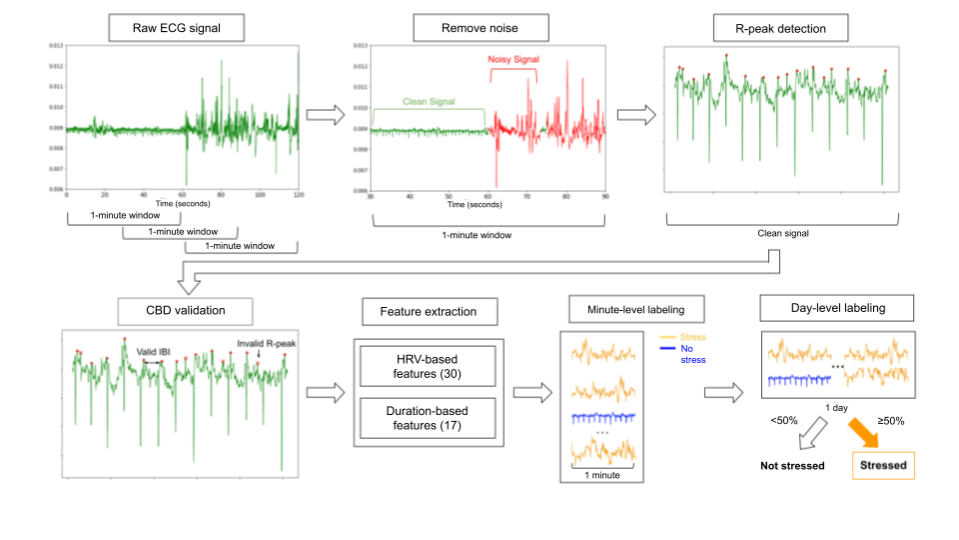
Sensor data processing pipeline. The red lines denote noisy segments of the signal by noise model. Red dots are R peaks, and red crosses are invalid peaks filtered by CBD. CBD: criterion beat difference; ECG: electrocardiography; HRV: heart rate variability; IBI: interbeat interval.

During the processing pipeline, we first filtered out noisy segments of the ECG signal and calculated interbeat intervals (IBIs) for each 1-minute segment. We then extracted HR variability (HRV)–based features and classified each minute as physiological stress positive or physiological stress negative (Figure 3).

To remove noisy signals caused by sensor deformation because of skin stretching, we first segmented the cleaned ECG signal using a window size of 1 minute with 30 seconds of overlap. Noise was filtered using an ensemble support vector machine (SVM) and neural network noise model described by Zhang et al [40]. The model involves further segmenting of each 1-minute ECG signal into 0.6-second intervals, extracting 3 HRV-based features from the R peaks detected, running both pretrained SVM and neural network classifiers, and classifying each interval as *clean* or *noisy* based on agreement between both models. Within each segment, we discarded segments with >20% noise. We further analyzed the cleaned 1-second segments using a reliability metric: the ratio of the number of data points collected in 1 second divided by the expected sampling rate. The segments were defined as high quality if the reliability was >80%, and low-quality segments were discarded.

Next, we repeated segmenting of clean ECG signals by 1-minute windows with 30 seconds of overlap to extract R peaks and IBIs. We then ran a Shannon energy‒based algorithm with modifications of nonlinear transformation and first-order Gaussian differentiator for extracting the initial set of R peaks [41,42]. Subsequently, we used the criterion beat difference [43] to filter out R peaks that were inconceivable (ie, out of normal heart rhythm range for humans). We then extracted timestamps between each pair of consecutive R peaks to calculate the IBIs.

#### Feature Extraction

A full list of features extracted for the model is provided in Table S2 in Multimedia Appendix 1.

Using the minute-level IBIs for each participant per day, we extracted 30 HRV-based features, and from these, we extracted 17 duration-based features. We calculated the average of each feature within a single day to define the day-level feature value. Given that prolonged stress may have different lasting effects compared with brief periods of physiological stress, we calculated an additional 17 duration-based features, which calculated the time spent physiologically stressed while wearing the sensor. As there is no formal duration that defines a *stressful event*, we crafted features that captured a range of minimum consecutive stress-positive window sizes (1, 2, 5, and 10 minutes). To create the features, we first adapted a pretrained SVM grid-search model from King et al [39] that classifies minute-level ECG signal as physiologically stress positive or physiologically stress negative. Specifically, the pretrained SVM model takes 30 statistical features extracted from R-R intervals (the time elapsed between 2 successive R-waves of the QRS signal on the electrocardiogram) and peaks by each minute of ECG signal and outputs the ground truth of stress positive or stress negative. Next, we used 1-, 2-, 5-, and 10-minute consecutive windows to derive total consecutive minutes and episodes from minute-level stress minutes.

We extracted 13 EMA-based features. A total of 12 questions were sent, with 7 questions inquiring about negative emotions and 5 questions inquiring about positive emotions (Table S1 in Multimedia Appendix 1).

These questions included the 4-item Perceived Stress Scale (PSS; ie, the PSS-4) [44], a widely used perceived stress evaluation questionnaire. The response options for each question of the PSS-4 range from 0 to 4, with the final range between 0 and 16, with 0 indicating no stress and 16 indicating very high stress. We calculated the mean score of the responses for each question per day to obtain the 12 daily scores as EMA-based features. By averaging responses to all the PSS-4 questions in a day, we derived the 13th EMA-based feature.

The following 4 intervention-based features were extracted from the 1:1 interventions and JIT interventions: intervention day (ie, whether the prior day was 1:1 intervention day), count intervention (cumulative number of 1:1 interventions received up to the prior day), JIT intervention day (ie, whether a JIT intervention was sent the prior day), and count JIT intervention (cumulative number of JIT interventions received up to the prior day). As the timing of intervention distribution varied, the *count JIT intervention* and *count intervention* variables enabled us to factor in the cumulative number of interventions. If either cumulative variable negatively predicted next-day stress, we were able to suggest that length of participation was negatively correlated with stress levels. If JIT intervention day and intervention day negatively predicted next-day stress, we were able to suggest short-term effectiveness of the interventions because they only indicated prior-day information.

We factored in the following 5 participant characteristics as covariates: age, gestational age at enrollment, number of prior pregnancies, number of prior children, and EPDS score (Table S3 in Multimedia Appendix 1 shows all participant characteristics).

#### Physiological Stress: Ground Truth

To establish ground truth for next-day physiological stress, we first fed the 30 HRV-based features to the model described by King et al [39] to determine the minute-level stress classification. From the model output, we calculated total consecutive stress minutes from all 1-minute–level classification results. A day was labeled as physiologically stress positive if the total number of consecutive stress minutes was >50%, based on previously published literature [45].

#### Perceived Stress: Ground Truth

Ground truth for perceived stress was labeled by calculating the average value of PSS-4 scores throughout a given day. PSS-4 was calculated using the Cohen Perceived Stress Scale by combining the 4 PSS items (PSS-Control, PSS-Overcome, PSS-Confident, and PSS–Your Way). A day was labeled as perceived stress positive if the PSS-4 score was >4.7 [46].

## Results

### Participants

A total of 16 pregnant women enrolled in the study. Demographics and data captured are shown in Table 1. In total, the participants collected 4157.18 hours of data over a total of 344 days, of which 256 (74.4%) were consecutive, with 114 (44.5%) being nonstressed days and 142 (55.5%) being stressed days. After filtering out noise, 89.2% (3708/4157.18) of the data remained clean for prediction. Participants wore the sensor for a mean of 21.5 (SD 5.21) days, with a mean of 16 (SD 3.28) days being consecutive days. Of the 16 participants, 14 (88%) had consecutive events of perceived stress recorded. Across all participants, a total of 956 days with EMA records were collected, with 881 (92.2%) being consecutive days. Among these 881 consecutive days, there were 412 days (46.7%) being nonstressed and 469 days (53.3%) being stressed. Participants answered a mean of 2.9 (SD 0.89) EMA questions per day. These results are supported by Wakschlag et al [45], who found that pregnant women experience stress an average of 49.9% of the day. Of the 16 participants, 3 (19%) did not wear the sensor for any consecutive days (necessary to predict next-day stress), and therefore data from these participants were discarded. Tables S4 and S5 in Multimedia Appendix 1 shows the quantity of sensor data and EMA data, respectively, collected by each participant.

**Table 1:** Participant characteristics and the amount of sensor and ecological momentary assessment (EMA) data captured (N=16).

Data captured			Values
Age (years), median (range)			35 (30-39)
Gestational age^a^ (weeks), median (range)			11.5 (10-17)
Number of prior children, median (range)			1 (0-2)
Number of prior pregnancies, median (range)			2 (1-5)
EPDS^b^ score, mean (SD)^a^			7.2 (3.4)
**Sensor data captured^c^**			
	Days worn, median (range)	23 (5-68)	
	Consecutive days worn, median (range)	16 (4-59)	
	Total wear time (hours), median (range)	245.6 (65.6-797.9)	
	Clean data (%), median (range)	87.9 (64.9-98.9)	
	Hours worn per day, mean (SD; range)	13.0 (1.55; 9.2-14.5)	
**EMA data captured^c^**			
	Days answered, median (range)	71 (30-94)	
	Consecutive days answered, median (range)	65.5 (24-93)	
	Total EMAs answered, median (range)	190 (64-346)	
	EMAs answered per day, mean (SD; range)	2.9 (0.88; 1.2-4.6)	

^a^At enrollment.

^b^EPDS: Edinburgh Postnatal Depression Scale.

^c^Data from 3 participants were outliers in wear time and thus excluded from analysis.

### Model Validation

#### Baseline Model Evaluation

We tested 6 widely used machine learning models using the scikit-learn Python package to evaluate the importance of input variables on next-day prediction of perceived and physiological stress [47-49]. In baseline models, we included the following models with default hyperparameters: gradient boost machine (min_samples_split: 5; min_samples_leaf: 2; max_depth: 3), SVM (kernel: rbf; C: 1.0; gamma: “scale”), adaptive boosting (n_estimators: 50), naïve Bayes, decision tree (min_samples_split: 5; min_samples_leaf: 2; max_depth: 3), and random forest (n_estimators: 10; min_samples_split: 5; min_samples_leaf: 2; max_depth: 3). In all baseline models, we used all 69 features as input to predict next day’s physiological and perceived stress and applied 5-fold cross-validation on each model. Each fold consisted of 80% training data and 20% testing data randomly selected from all participants combined. We adopted commonly used evaluation metrics (precision, recall, and F1 score) for binary classification [50]. The random forest classifier performed best across both types of stress, with an average F1 score of 81.9% when predicting physiological stress and 72.5% when predicting perceived stress.

#### Correlation-Based Feature Subset Selection

We used correlation-based feature selection (CFS) [51] on our set of 69 features (4 intervention related, 5 covariates, 30 HRV based, 17 duration based, and 13 EMA based). CFS helps evaluate the intrinsic correlations within features to avoid redundancy and high feature-class correlation to maintain or increase predictive power. CFS helped to reduce the number of features, which allowed us to understand which data we may not need to collect in the future or to explain which features are not contributing to the resulting prediction.

#### Bayesian Optimization

In addition to CFS for selecting the optimal subset of features, we adapted Bayesian optimization based on the work of Snoek et al [52] and the Python implementation package built by Nogueira [53]. In Bayesian optimization, the general performance of the selected machine learning algorithms was modeled as a sample from a Gaussian process, and the nature of the Gaussian process helped to optimize and tune the hyperparameters to further improve the model performance.

### Combination of Feature Types

**Figure 4:**
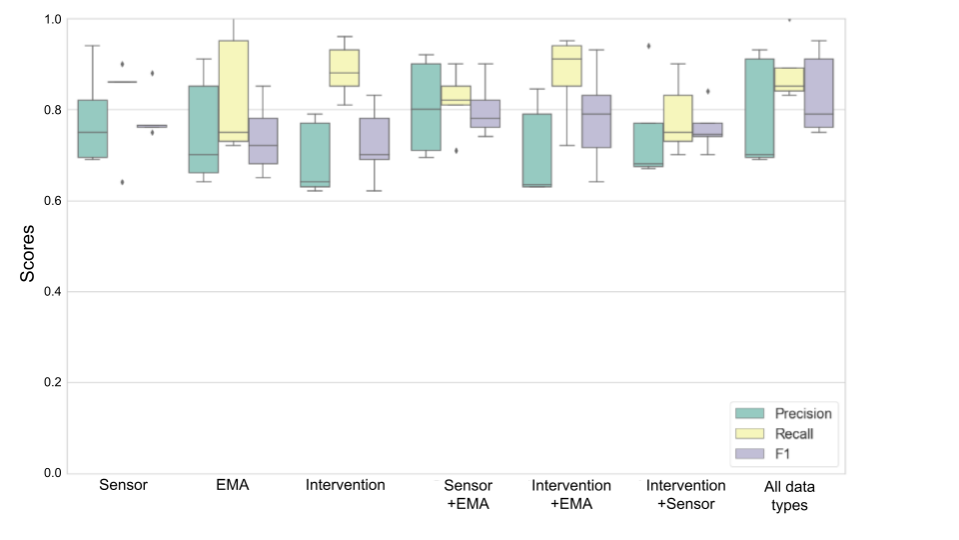
5-fold cross-validation of next-day physiological stress by subset of feature types. EMA: ecological momentary assessment.

**Figure 5:**
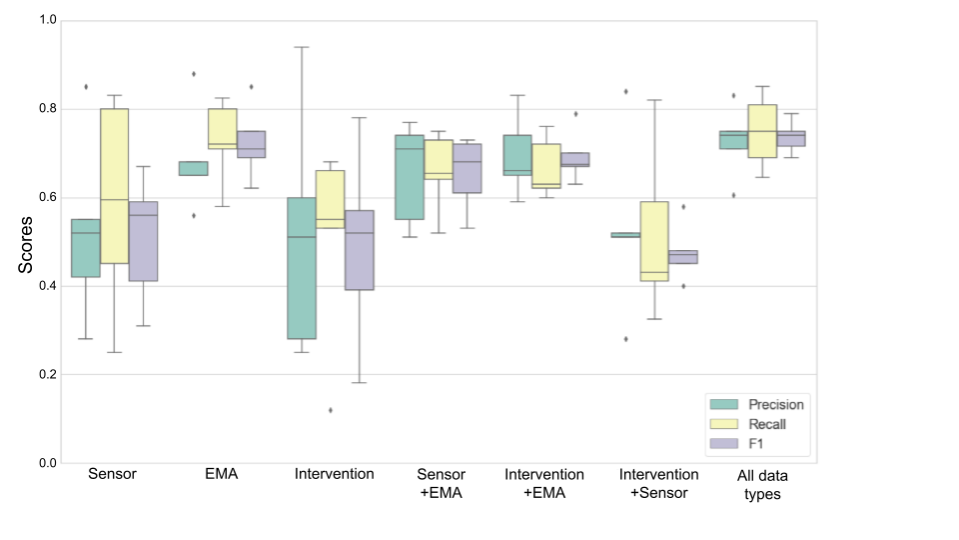
5-fold cross-validation of next-day perceived stress by subset of feature types. EMA: ecological momentary assessment.

To reduce the burden of data collection by removing features without sacrificing significant predictive power (as measured by the F1 score), we ran random forest with 5-fold cross-validation with various combinations of sensor-, EMA-, and intervention-based data. For both types of stress, we used 6 combinations of data: sensor only, EMA only, intervention only, sensor with EMA, intervention with EMA, and intervention with sensor. We then compared the results with those of a model that used all types of data. For physiological stress predictions, with any combination of data types, the average F1 score remained >73% (Figure 4). For perceived stress predictions, only combinations with EMA data continued to perform well (Figure 5).

### Model Performance

First, CFS was applied to select the subset of features used to build physiological and perceived stress models. Next, we applied Bayesian optimization to all the baseline models. Random forest outperformed the rest of the models after the hyperparameters were optimized (n_estimators, criterion, max_depth, min_samples_split, and max_features) using a range of 200 values for each continuous hyperparameter (eg, n_estimators) and the maximum number of options for each categorical hyperparameter (eg, criterion). The detailed hyperparameters after optimization are presented in Table S6 in Multimedia Appendix 1. The resulting F1 score increased to 83.6% when predicting physiological stress and 74.4% when predicting perceived stress.

**Figure 6:**
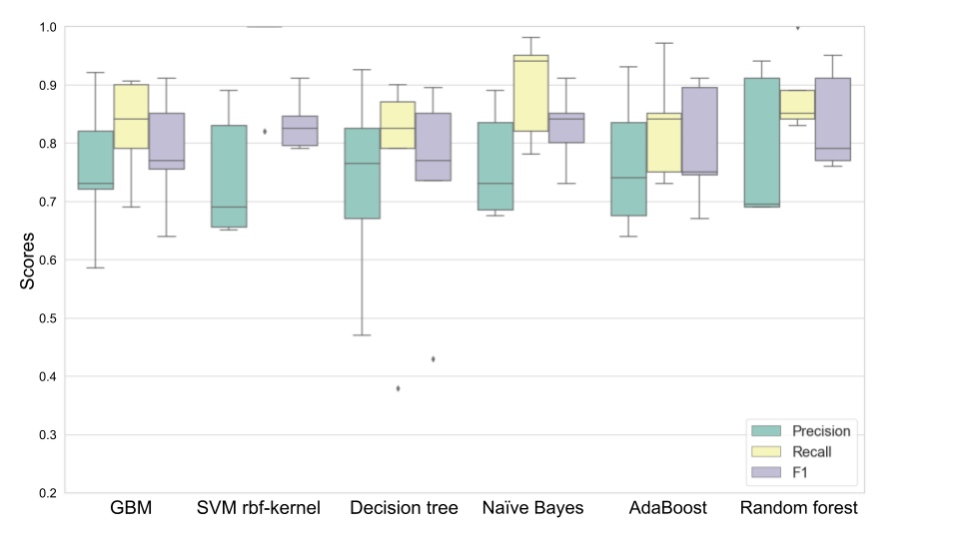
Predicting next-day physiological stress by using 6 different machine learning models using 5-fold cross-validation. Boxes indicate the IQR, whiskers indicate the minimum and maximum, and solid lines indicate the median. AdaBoost: adaptive boosting; GBM: gradient boosting machine; SVM: support vector machine.

**Figure 7:**
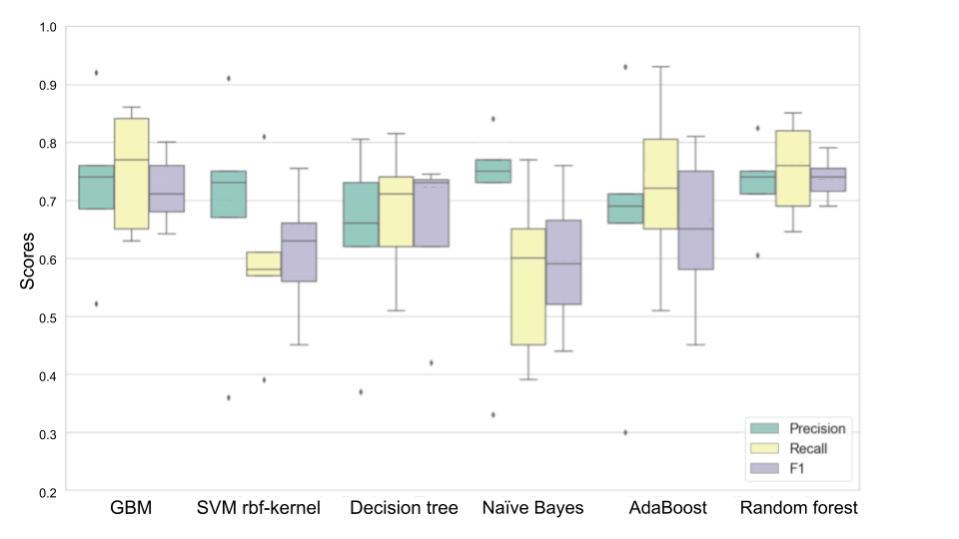
Predicting next-day physiological stress by using 6 different machine learning models using 5-fold cross-validation. Boxes indicate the IQR, whiskers indicate the minimum and maximum, and solid lines indicate the median. AdaBoost: adaptive boosting; GBM: gradient boosting machine; SVM: support vector machine.

Figures 6 and 7 show the results of using the 6 different classifiers to predict next-day physiological stress and perceived stress, respectively, and show F1 scores in descending order using the associated subset of features identified by CFS. These data in table format are shown in Tables S7 and S8 in Multimedia Appendix 1.

### Feature Importance

**Figure 8:**
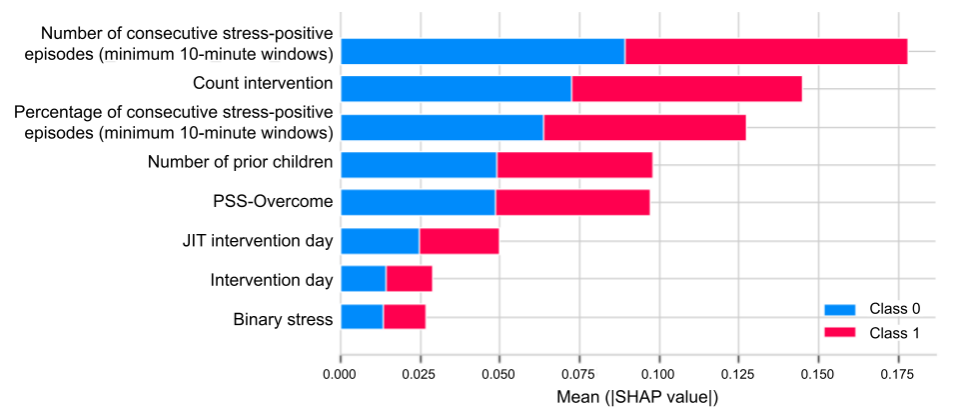
SHAP summary plot for feature importance in predicting physiological stress. JIT: just-in-time; PSS: Perceived Stress Scale; SHAP: Shapley Additive Explanations.

We applied SHAP on the 8 features selected by the random forest model (because random forest performed best with the highest F1 score) to predict physiological stress. The top 5 features ranked by mean absolute SHAP values were as follows: number of consecutive stress minutes by 10-minute minimum threshold, count of interventions, number of consecutive stress minutes percentage by 10-minute minimum threshold, number of children, and PSS-Overcome (Figure 8). The following were also predictive but exhibited mean SHAP values of <0.05, suggesting lower predictive value: JIT intervention day, intervention day, and binary stress.

**Figure 9:**
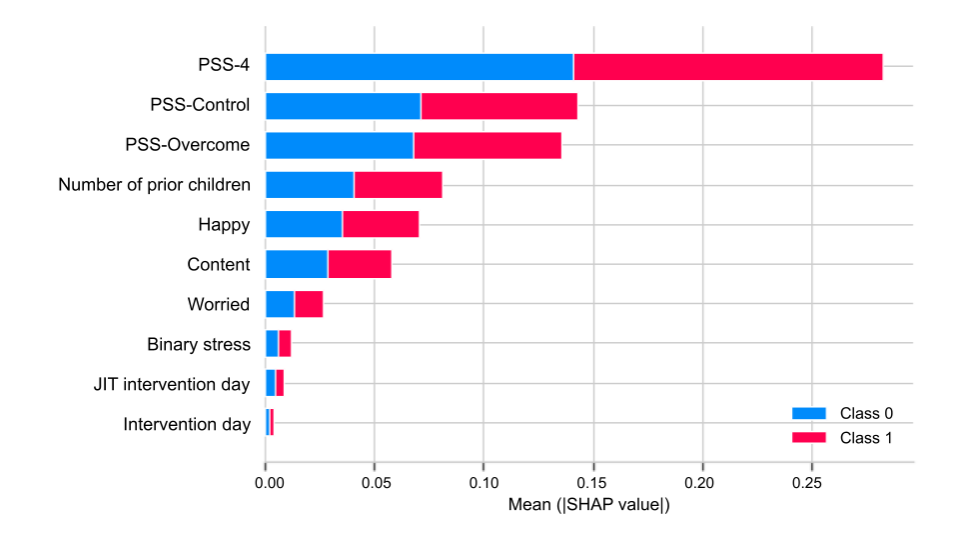
SHAP summary plot for feature importance in predicting perceived stress. JIT: just-in-time; PSS: Perceived Stress Scale; SHAP: Shapley Additive Explanations.

Similarly, SHAP was applied on 10 features selected by the random forest model to predict perceived stress. The top 6 features ranked by mean absolute SHAP values were as follows: PSS-4, PSS-Control, PSS-Overcome, number of children, happy stress, and content stress (Figure 9). The following features were also predictive but exhibited mean SHAP values of <0.05: worried stress, binary stress, JIT intervention day, and intervention day.

**Figure 10:**
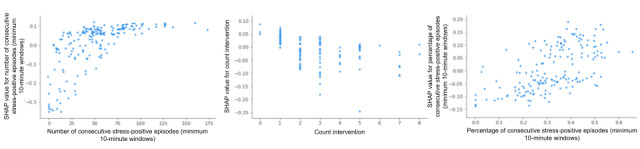
SHAP dependence plot for physiological stress features with values >0.05. SHAP: Shapley Additive Explanations.

The SHAP analysis of physiological stress (Figure 10) showed that the greater the number of consecutive stress episodes (minimum of 10 minutes per stress event), the more likely the following day would also be a physiologically stressful day. Conversely, the greater the number of count interventions (cumulative number of 1:1 interventions received up to the prior day), the lower the physiological stress the next day.

**Figure 11:**
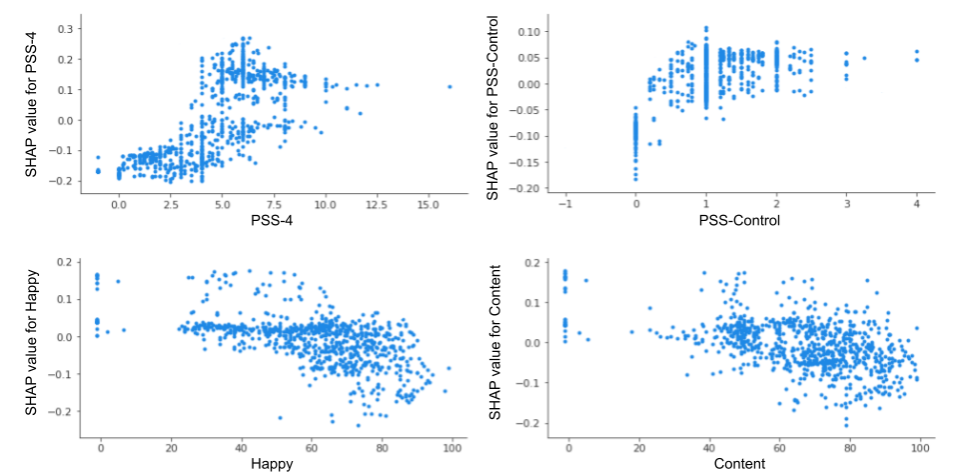
SHAP dependence plot for perceived stress features with values >0.05. PSS: Perceived Stress Scale; SHAP: Shapley Additive Explanations.

The SHAP analysis of perceived stress (Figure 11) shows that low values of PSS-4 (<4.0) are characterized by negative SHAP values; this suggests that lower PSS-4 scores are related to a lower likelihood of next-day perceived stress prediction. High values of PSS-Control (>2.0; ie, not feeling as though one can control important things) were generally associated with positive SHAP values: predictions of higher perceived stress the next day.

Furthermore, we observed a distribution of negative SHAP values for happy stress scores >20 and content stress scores >40, suggesting that higher scores in these areas tend to drive predictions of less stress the following day. However, some observations with positive SHAP values were distributed across a wide range of scores; we suspect these were due to interactions with other features.

**Figure 12:**
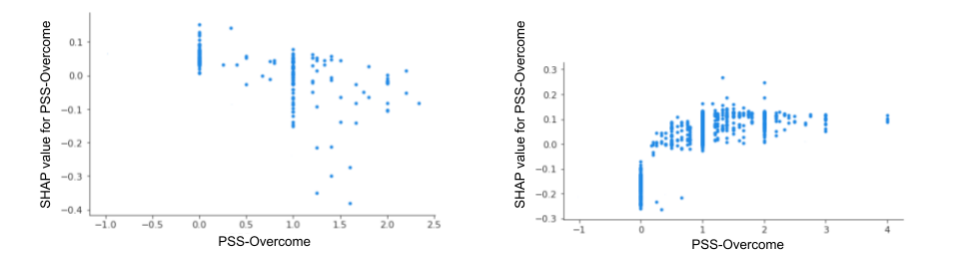
SHAP dependence plot for the shared physiological stress (left plot) and perceived stress (right plot) feature PSS-Overcome. PSS: Perceived Stress Scale; SHAP: Shapley Additive Explanations.

**Figure 13:**
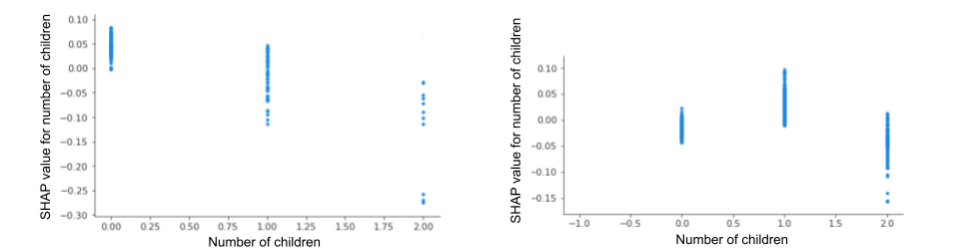
SHAP dependence plot for the shared physiological stress (left plot) and perceived stress feature (right plot) number of children. SHAP: Shapley Additive Explanations.

The EMA-based feature PSS-Overcome (“Did you feel difficulties piling up so you cannot overcome them?”) generally seemed to predict lower levels of next-day physiological stress but higher levels of next-day perceived stress (Figure 12). Although the feature *number of children* scored highly important when predicting next-day physiological stress and perceived stress (Figures 8 and 9, respectively), according to the summary plot (Figure 13), we see variability in how the number of children a mother has influenced physiological and perceived stress. Physiologically, having no child (and therefore being pregnant with a first child) is positively associated with an increase in physiological stress and having 2 children (and therefore being pregnant with a third child) is associated with a reduced probability of physiological stress for the next day. Perceptually, it seems that the first time a mother is pregnant while caring for her first child, she experiences greater stress than when she has no existing children or has already gone through the experience.

### User Feedback

**Figure 14:**
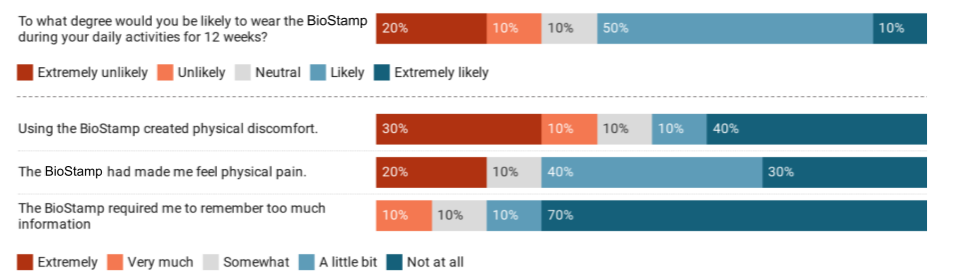
Participants’ feedback regarding BioStampRC usability and wearability.

Of the 14 participants, 10 (71%) completed the full feedback survey on wearability and usability of the sensor (Figure 14). Most (7/10, 70%) of the respondents reported that the device was not painful; however, 20% (2/10) reported extreme pain because of the accompanying strong adhesive of the device and repeated application to the same location every day. When considering physical discomfort when the device was worn, 50% (5/10) of the respondents reported discomfort to be *a little bit* or *not at all*, whereas 50% (5/10) reported discomfort to be ranging from *somewhat* to *extreme*. Most (8/10, 80%) of the respondents found the device easy to use.

**Figure 15:**
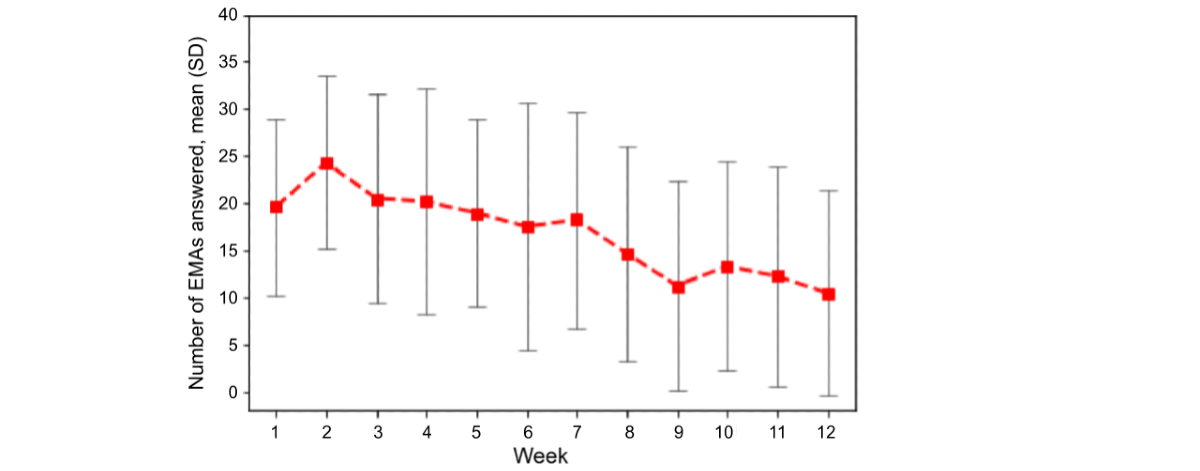
Average response rate to daily EMA surveys per study week. EMA: ecological momentary assessment.

To measure the burden of self-report, we hypothesized that frequent EMA surveys were burdensome to participants in longitudinal studies [32] and because of burnout response rates would diminish as participants continued in the study. In this study, participant responsiveness peaked at the second week and continuously decreased throughout the following weeks, with the lowest response rates in the last week of the study (Figure 15). In addition, many participants reported concerns about the frequency of the EMAs. Of the 10 participants who completed the survey, 4 (40%) stated that EMAs were sent too often and 2 (20%) stated that the timing was not always convenient, which may have been because of sending multiple surveys that were ≥2 hours apart in a day.

## Discussion

### Principal Findings

A total of 16 pregnant women enrolled in our pilot study for predicting next-day stress in pregnant women participating in a CBT-based course aimed at reducing stress. Overall, 4157.18 hours of data were collected and participants answered 2838 EMAs. Approximately half (142/256, 55.5%) of the days were determined to be stressed days, which is in line with the results from Wakschlag et al [45], who found that pregnant women experience stress an average of 49.9% of the day. In our study, we identified sensor-based features that best predicted next-day physiological stress and EMA-based features that best predicted next-day perceived stress. Notably, 2 features emerged for predicting both physiological stress and perceived stress: the EMA question PSS-Overcome and the number of children the participant already had.

Our results inform opportunities and challenges with using various measures to predict perceived and physiological stress 1 day in advance and highlight the temporal and relational differences between perceived stress and physiological stress. The significant input variables noted provide opportunity for predictive systems, including machine learning models, to be tailored using these variables in scheduling future interventions. For instance, perhaps sensor data should not be used to predict next-day perceived stress, which can be better predicted using EMA data, the low-burden intervention-based data, or covariates.

### Explaining Important Features in Predicting Next-Day Physiological Stress

After feature reduction using only sensor-based data with covariates (average F1 score of 78.3%), all the important features were consistently related to sustained attributes: number of consecutive stress episodes of a minimum duration of 10 minutes, number of children, percentage of wear time episodes spent stress positive (10-minute minimum threshold), number of prior pregnancies, gestation week, age, and depression score. Most features identified to be predictive are not prone to quick change, suggesting that the carryover of physiological stress can be a reflection of chronic stress.

The features with a SHAP value >0.05 that predicted next-day physiological stress were as follows: number of consecutive stress episodes of a minimum duration of 10 minutes, intervention count, number of consecutive perceived stress episodes of a minimum duration of 10 minutes, number of children, and the EMA question PSS-Overcome. Overall, the greater the percentage of episodes classified as stress positive, the more likely the following day was classified as stress positive. This suggests that when there is prolonged physiological stress during a day, it is more likely that the next day will continue to be physiologically stressful. This could also indicate that a minimum duration of 5 minutes is required to reduce the influence of false positives and capture more substantial episodes of stress.

The high feature importance of count intervention, or the cumulative number of interventions the participant received, further suggests the lasting effects of physiological stress and interventions. The more interventions the participant received, the more likely they were to have lower stress the following day up until 4 interventions were received, at which point the effect flattened. A randomized controlled trial would be useful to distinguish whether the interventions were actually effective in lowering next-day stress and whether the mindfulness-based skills were effective in lowering physiological stress. Moreover, in-person visits are costly and affect the scalability of the intervention. Our findings suggest that there may be an optimal number of in-person visits needed to reduce stress. Further research may aim to compare the effects of adjusting the number of in-person visits and its effects on cost and physiological stress reduction.

### Explaining Important Features in Predicting Next-Day Perceived Stress

Similar to our analysis of physiological stress, we analyzed the dependencies of features that predicted next-day perceived stress based on SHAP mean values >0.05. The highly predictive features were as follows: PSS-4, PSS-Control, PSS-Overcome, number of children, happy stress, and content stress. Our results show that a participant’s perceived ability to control important things were predictive of perceived stress the following day, whereas happy stress and content stress were predictive of lower perceived stress the following day. These results suggest that there is a lasting effect of perceived stress into the following day. The findings that perceived stress can carry over because it may linger in the mind has been identified in previous studies [54], and our findings confirm that this applies to pregnant women. In addition, when studying the different combinations of variable types for their ability to predict next-day perceived stress, we found that models that excluded EMA features performed poorly, suggesting that, unlike prediction of next-day physiological stress, alternative data collection methods do not replace EMA features. However, our user feedback survey showed that participants considered the EMAs in our study to be burdensome, confirming the need for lower-burden self-report models for detection and prediction of stress; thus, future work must confirm the most predictive features of next-day stress before incorporating them into stress-reducing interventions to ensure that the intervention does not lead to excess user burden and thus increased stress.

In terms of next-day perceived stress, increased perceptions of stress can carry over to the following day, as shown in our prior findings and in previous literature [54]. However, it was surprising that, generally, higher values of PSS-Overcome were associated with lower probabilities of next-day physiological stress. A prior study showed a moderate positive correlation (*r*=0.48) between intended in-laboratory stressors and PSS-Overcome [39]. Our findings suggest that PSS-Overcome may have a reverse predictive value over time (ie, the next day). Future research should investigate the predictive value of these features over time.

### Number of Children and Feeling Unable to Overcome Difficulties

The covariate number of children and the EMA question PSS-Overcome were ranked as highly important in predicting next-day physiological and perceived stress but in opposing valence. This reinforces the narrative that physiological stress and perceived stress are conceptually and temporally divergent. Our results suggest that generally, for any given day, the more children a participant had, the lower their physiological stress was predicted to be the following day. This may be explained by the initial stressful transition into motherhood and gradual acclimation or accumulation of resilience with each new child. Simultaneously, when it comes to predicting next-day perceived stress, participants about to have their second child were generally more likely to feel stressed the following day. Recent research suggests that having a second child does worsen parents’ mental health, in particular for women who often bear the brunt of child-rearing tasks [55]. Our results suggest that parents often do not expect that the work in raising a second child would be exponentially greater after already having a child. However, a drop in next-day perceived stress during a third pregnancy may signify that coping strategies for handling pregnancy and an additional child are learned. These findings offer a unique perspective into physiological and perceived experiences of stress through a cross-section of stages throughout the journey of parenthood. Future studies are needed to verify our findings through a longitudinal study.

### Future of Mobile Health Systems in Mental Health Prediction and Intervention

Future work in developing mobile health systems that detect physiological and perceived incidences of problematic mental health episodes should investigate and compare the predictive value of sensor-captured data, self-reported measures, and other incidentally captured data through means such as intervention schedules and covariates. In this study, we investigated 2 types of chronic stress: perceived stress and chronic strain, which most consistently predict PD, making it an appropriate target for PD prevention both psychologically and physiologically [1]. Using a multimodal detection system allowed us to not only identify features that most strongly predicted physiological and perceived stress but also allowed us to discover how to minimize the features that are necessary to make next-day predictions. Other developers of mobile health systems for mental health detection may also consider identifying how specific episodes manifest physiologically and perceptually. Ultimately, to create an effective predictive mobile health system with JIT interventions, each component—sensor, EMA, and intervention—must be sustainable and usable.

The accompanying strong adhesive used in the sensors for physiological stress detection was reported by 20% (2/10) of the participants to cause extreme physical pain because of repeated application to the same location every day, which is a barrier to creating such a sustainable system. Prolonged wear and comfort are critical because our findings highlight the importance of predicting 10-minute bouts of physiological stress through sensor data as potential indicators of chronic strain that is likely to persist the following day. In addition, short battery lifetime is a barrier to extended wear. To collect more robust data, increasing battery lifetime and sensor size may stand in opposition to the comfort of participants. Future work should investigate finding a balance between the sensor’s robustness and user willingness to wear it.

In the absence of costly and possibly uncomfortable sensors, EMAs were a pathway to predicting next-day perceived or physiological stress. However, responsiveness to EMAs decreased after the first 2 weeks. Studies have shown that user-friendly interfaces and directly useful features such as allowing the data to be viewable to participants and increasing their self-awareness and tracking their progress can increase engagement with EMAs [56,57]. Creating a sustainable system that incorporates the collection of perceived mental health status will require directly providing more value to users. Furthermore, as perceived stress is malleable to psychological intervention whereas chronic stress is not, perceived stress is a viable intervention target [45,58].

Incidentally captured data in the form of interventions, covariates, or other nonsensor passively captured data offer additional opportunity to predict next-day mental health concerns. This category of features is low burden for individuals to collect and may still be strong predictors. These data may also offer more data in the form of contextualization to create a robust system of sensing and intervention at the most opportune times [59,60]. For example, knowing the timing and effectiveness of a recently completed intervention may allow a system to recommend related interventions in the future.

### Limitations

To learn about the feasibility of wearing an ECG sensor longitudinally, we conducted a study in a natural setting, but this presents unavoidable natural variations in wear time. For instance, over the 12-week period when participants wore the device, the average wear time was 11.5 hours per day. Although most participants found the device easy to use, albeit somewhat painful (because of the repeated application of the strong adhesive in the same location), the disagreement between perceived stress and physiological stress could be a consequence of participants not wearing the device specifically during stressful moments of the day. Although minutes-ahead predictors may hold for short-term studies, challenges with wearability [61] and sensor quality [62] over time will still likely affect prediction accuracy. Our analysis is limited by the distribution of the participants’ ages, which were in the range of 30 to 39 years. Although this was not intentional, it allowed us to collect data from a distribution of first-time pregnancies and non–first-time pregnancies. However, these data do not reflect mothers who may have had children earlier or later in life.

### Conclusions

In this work, we used machine learning and SHAP to predict and explain the relationships between potential predictors of next-day physiological stress and perceived stress. We built interpretable models to predict next-day physiological stress with an F1 score of 83.6% and next-day perceived stress with an F1 score of 74.4%. We further identified unique features such as number and percentage of consecutive stress episodes of a minimum duration of 10 minutes to be predictive of next-day physiological stress. Using this technique, we evaluated the feature space of intervention-, sensor-, and EMA-based data to find features that can predict next-day physiological stress and perceived stress. Our results show that it is possible to predict next-day physiological and perceived stress while reducing the burden of data collection. Our study is the first of its kind in terms of assessing pregnant women over a period of 12 weeks (vs a single day in the study by King et al [39]); however, future studies should validate our models with a larger participant sample. Although tomorrow’s stress is imminent, future stress research should consider predicting further future stress at other time points such as the following week to understand the sustained predictive value of these features.

## Appendix

Multimedia Appendix 1 Supplementary figure and tables.

## Abbreviations

CBT: cognitive behavioral therapy
CFS: correlation-based feature selection
ECG: electrocardiography
EMA: ecological momentary assessment
EPDS: Edinburgh Postnatal Depression Scale
HR: heart rate
HRV: heart rate variability
IBI: interbeat interval
JIT: just-in-time
MB: Mothers and Babies
PD: perinatal depression
PSS: Perceived Stress Scale
SHAP: Shapley Additive Explanations
SVM: support vector machine

## References

[1] Yim IS, Tanner Stapleton LR, Guardino CM, Hahn-Holbrook J, Dunkel Schetter C (2015). Biological and psychosocial predictors of postpartum depression: systematic review and call for integration. Annu Rev Clin Psychol.

[2] McCormick MC, Brooks-Gunn J, Shorter T, Holmes JH, Wallace CY, Heagarty MC (1990). Factors associated with smoking in low-income pregnant women: relationship to birth weight, stressful life events, social support, health behaviors and mental distress. J Clin Epidemiol.

[3] Satyanarayana VA, Lukose A, Srinivasan K (2011). Maternal mental health in pregnancy and child behavior. Indian J Psychiatry.

[4] Glynn LM, Schetter CD, Hobel CJ, Sandman CA (2008). Pattern of perceived stress and anxiety in pregnancy predicts preterm birth. Health Psychol.

[5] Lobel M, Cannella DL, Graham JE, DeVincent C, Schneider J, Meyer BA (2008). Pregnancy-specific stress, prenatal health behaviors, and birth outcomes. Health Psychol.

[6] Hoyert DL, Xu J (2012). Deaths: preliminary data for 2011. Natl Vital Stat Rep.

[7] Xu J, Murphy SL, Kochanek KD, Bastian B, Arias E (2018). Deaths: final data for 2016. Natl Vital Stat Rep.

[8] Mulder EJ, Robles de Medina PG, Huizink AC, Van den Bergh BR, Buitelaar JK, Visser GH (2002). Prenatal maternal stress: effects on pregnancy and the (unborn) child. Early Hum Dev.

[9] Nimby GT, Lundberg L, Sveger T, McNeil TF (1999). Maternal distress and congenital malformations: do mothers of malformed fetuses have more problems?. J Psychiatr Res.

[10] Beddoe AE, Paul Yang CP, Kennedy HP, Weiss SJ, Lee KA (2009). The effects of mindfulness-based yoga during pregnancy on maternal psychological and physical distress. J Obstet Gynecol Neonatal Nurs.

[11] Carolan M, Barry M, Gamble M, Turner K, Mascareñas O (2012). The Limerick Lullaby project: an intervention to relieve prenatal stress. Midwifery.

[12] Duncan LG, Bardacke N (2010). Mindfulness-based childbirth and parenting education: promoting family mindfulness during the perinatal period. J Child Fam Stud.

[13] Dunn C, Hanieh E, Roberts R, Powrie R (2012). Mindful pregnancy and childbirth: effects of a mindfulness-based intervention on women's psychological distress and well-being in the perinatal period. Arch Womens Ment Health.

[14] Guardino CM, Dunkel Schetter C, Bower JE, Lu MC, Smalley SL (2014). Randomised controlled pilot trial of mindfulness training for stress reduction during pregnancy. Psychol Health.

[15] Muzik M, Hamilton SE, Lisa Rosenblum K, Waxler E, Hadi Z (2012). Mindfulness yoga during pregnancy for psychiatrically at-risk women: preliminary results from a pilot feasibility study. Complement Ther Clin Pract.

[16] Dennis CL, Dowswell T (2013). Psychosocial and psychological interventions for preventing postpartum depression. Cochrane Database Syst Rev.

[17] Hofmann SG, Asnaani A, Vonk IJ, Sawyer AT, Fang A (2012). The efficacy of cognitive behavioral therapy: a review of meta-analyses. Cognit Ther Res.

[18] O'Connor E, Senger CA, Henninger ML, Coppola E, Gaynes BN (2019). Interventions to prevent perinatal depression: evidence report and systematic review for the US Preventive Services Task Force. JAMA.

[19] Tandon SD, Ward EA, Hamil JL, Jimenez C, Carter M (2018). Perinatal depression prevention through home visitation: a cluster randomized trial of mothers and babies 1-on-1. J Behav Med.

[20] Kazantzis N, L'Abate L (2007). Handbook of Homework Assignments in Psychotherapy: Research, Practice, and Prevention.

[21] Hardeman W, Houghton J, Lane K, Jones A, Naughton F (2019). A systematic review of just-in-time adaptive interventions (JITAIs) to promote physical activity. Int J Behav Nutr Phys Act.

[22] Firth J, Torous J, Nicholas J, Carney R, Rosenbaum S, Sarris J (2017). Can smartphone mental health interventions reduce symptoms of anxiety? A meta-analysis of randomized controlled trials. J Affect Disord.

[23] Heron KE, Smyth JM (2010). Ecological momentary interventions: incorporating mobile technology into psychosocial and health behaviour treatments. Br J Health Psychol.

[24] Proudfoot J, Parker G, Hadzi Pavlovic D, Manicavasagar V, Adler E, Whitton A (2010). Community attitudes to the appropriation of mobile phones for monitoring and managing depression, anxiety, and stress. J Med Internet Res.

[25] Smyth JM, Heron KE (2016). Is providing mobile interventions "just-in-time" helpful? An experimental proof of concept study of just-in-time intervention for stress management. Proceedings of the 2016 IEEE Wireless Health.

[26] Goodday SM, Friend S (2019). Unlocking stress and forecasting its consequences with digital technology. NPJ Digit Med.

[27] Sapolsky RM (1996). Why stress is bad for your brain. Science.

[28] von Känel R (2015). Acute mental stress and hemostasis: when physiology becomes vascular harm. Thromb Res.

[29] Hamarat E, Thompson D, Zabrucky KM, Steele D, Matheny KB, Aysan F (2001). Perceived stress and coping resource availability as predictors of life satisfaction in young, middle-aged, and older adults. Exp Aging Res.

[30] Jaimes LG, Llofriu M, Raij A (2014). A stress-free life: just-in-time interventions for stress via real-time forecasting and intervention adaptation. Proceedings of the 9th International Conference on Body Area Networks.

[31] Munoz-Organero M, Corcoba-Magana V (2017). Predicting upcoming values of stress while driving. IEEE Trans Intell Transport Syst.

[32] Musthag M, Raij A, Ganesan D, Kumar S, Shiffman S (2011). Exploring micro-incentive strategies for participant compensation in high-burden studies. Proceedings of the 13th International Conference on Ubiquitous Computing.

[33] Sarsenbayeva Z, van Berkel N, Hettiachchi D, Jiang W, Dingler T, Velloso E, Kostakos V, Goncalves J (2019). Measuring the effects of stress on mobile interaction. Proc ACM Interact Mob Wearable Ubiquitous Technol.

[34] Rozet A, Kronish IM, Schwartz JE, Davidson KW (2019). Using machine learning to derive just-in-time and personalized predictors of stress: observational study bridging the gap between nomothetic and ideographic approaches. J Med Internet Res.

[35] Umematsu T, Sano A, Taylor S, Picard R (2019). Improving students' daily life stress forecasting using LSTM neural networks. Proceedings of the 2019 IEEE International Conference on Biomedical and Health Informatics.

[36] Lundberg SM, Lee SI (2017). A unified approach to interpreting model predictions. Proceedings of the 31st International Conference on Neural Information Processing Systems.

[37] Le HN, Perry DF, Mendelson T, Tandon SD, Muñoz RF (2015). Preventing perinatal depression in high risk women: moving the mothers and babies course from clinical trials to community implementation. Matern Child Health J.

[38] Barrera AZ, Hamil J, Tandon D (2021). Integrating SMS text messages into a preventive intervention for postpartum depression delivered via in-home visitation programs: feasibility and acceptability study. JMIR Form Res.

[39] King ZD, Moskowitz J, Egilmez B, Zhang S, Zhang L, Bass M, Rogers J, Ghaffari R, Wakschlag L, Alshurafa N (2019). Micro-stress EMA: a passive sensing framework for detecting in-the-wild stress in pregnant mothers. Proc ACM Interact Mob Wearable Ubiquitous Technol.

[40] Zhang L, King Z, Egilmez B, Reeder J, Ghaffari R, Rogers J, Rosen K, Bass M, Moskowitz J, Tandon D, Wakschlag L, Alshurafa N (2018). Measuring fine-grained heart-rate using a flexible wearable sensor in the presence of noise. Proceedings of the IEEE 15th International Conference on Wearable and Implantable Body Sensor Networks.

[41] Kathirvel P, Sabarimalai Manikandan M, Prasanna SR, Soman KP (2011). An efficient R-peak detection based on new nonlinear transformation and first-order Gaussian differentiator. Cardiovasc Eng Tech.

[42] Manikandan MS, Soman KP (2012). A novel method for detecting R-peaks in electrocardiogram (ECG) signal. Biomed Signal Process Control.

[43] Berntson GG, Quigley KS, Jang JF, Boysen ST (1990). An approach to artifact identification: application to heart period data. Psychophysiology.

[44] Cohen S (1994). Perceived stress scale. Mind Garden.

[45] Wakschlag LS, Tandon D, Krogh-Jespersen S, Petitclerc A, Nielsen A, Ghaffari R, Mithal L, Bass M, Ward E, Berken J, Fareedi E, Cummings P, Mestan K, Norton ES, Grobman W, Rogers J, Moskowitz J, Alshurafa N (2021). Moving the dial on prenatal stress mechanisms of neurodevelopmental vulnerability to mental health problems: a personalized prevention proof of concept. Dev Psychobiol.

[46] Cohen S, Kamarck T, Mermelstein R (1983). A global measure of perceived stress. J Health Soc Behav.

[47] Chung H, Ko H, Kang WS, Kim KW, Lee H, Park C, Song HO, Choi TY, Seo JH, Lee J (2021). Prediction and feature importance analysis for severity of COVID-19 in South Korea using artificial intelligence: model development and validation. J Med Internet Res.

[48] Jaques N, Taylor S, Azaria A, Ghandeharioun A, Sano A, Picard R (2015). Predicting students' happiness from physiology, phone, mobility, and behavioral data. Int Conf Affect Comput Intell Interact Workshops.

[49] Sang S, Sun R, Coquet J, Carmichael H, Seto T, Hernandez-Boussard T (2021). Learning from past respiratory infections to predict COVID-19 outcomes: retrospective study. J Med Internet Res.

[50] Ferrario A, Demiray B, Yordanova K, Luo M, Martin M (2020). Social reminiscence in older adults' everyday conversations: automated detection using natural language processing and machine learning. J Med Internet Res.

[51] Hall MA (1999). Correlation-based feature selection for machine learning. University of Waikato.

[52] Snoek J, Larochelle H, Adams RP (2012). Practical Bayesian optimization of machine learning algorithms. Proceedings of the Advances in Neural Information Processing Systems 25.

[53] Nogueira F (2014). Bayesian Optimization: Open source constrained global optimization tool for Python. GitHub.

[54] Hovsepian K, Al'Absi M, Ertin E, Kamarck T, Nakajima M, Kumar S (2015). cStress: towards a gold standard for continuous stress assessment in the mobile environment. Proceedings of the 2015 ACM International Joint Conference on Pervasive and Ubiquitous Computing.

[55] Ruppanner L, Perales F, Baxter J (2019). Harried and unhealthy? Parenthood, time pressure, and mental health. J Marriage Fam.

[56] Hartmann R, Sander C, Lorenz N, Böttger D, Hegerl U (2019). Utilization of patient-generated data collected through mobile devices: insights from a survey on attitudes toward mobile self-monitoring and self-management apps for depression. JMIR Ment Health.

[57] Ortiz A, Grof P (2016). Electronic monitoring of self-reported mood: the return of the subjective?. Int J Bipolar Disord.

[58] Brown H, Krogh-Jespersen S, Tandon D, Graham A, Mackiewicz Seghete K, Wakschlag L, Wazana A, Székely E, Oberlander TF (2021). Looking ahead: pre-and perinatal interventions for maternal distress to prevent neurodevelopmental vulnerability. Prenatal Stress and Child Development.

[59] Liao P, Dempsey W, Sarker H, Hossain SM, Al'absi M, Klasnja P, Murphy S (2018). Just-in-time but not too much: determining treatment timing in mobile health. Proc ACM Interact Mob Wearable Ubiquitous Technol.

[60] Mishra V, Künzler F, Kramer JN, Fleisch E, Kowatsch T, Kotz D (2021). Detecting receptivity for mHealth interventions in the natural environment. Proc ACM Interact Mob Wearable Ubiquitous Technol.

[61] Sarker H, Hovsepian K, Chatterjee S, Nahum-Shani I, Murphy S, Spring B, Rehg JM, Murphy SA, Kumar S (2017). From markers to interventions: the case of just-in-time stress intervention. Mobile Health: Sensors, Analytic Methods, and Applications.

[62] Evenson KR, Goto MM, Furberg RD (2015). Systematic review of the validity and reliability of consumer-wearable activity trackers. Int J Behav Nutr Phys Act.

