# Supplementary material for: Predicting the Next-Day Perceived and Physiological Stress of Pregnant Women by Using Machine Learning and Explainability: Algorithm Development and Validation
Source: JMIR Mhealth Uhealth. 2022 Aug 2;10(8):e33850. doi: 10.2196/33850 (PMC9382551; doi:10.2196/33850)
Supplement: Multimedia Appendix 1 [file mhealth_v10i8e33850_app1.docx]

# Supplementary Materials

Supplementary Figure 1. ECG sensor given to participants and its paired tablet for controlling sensor recording and data upload.


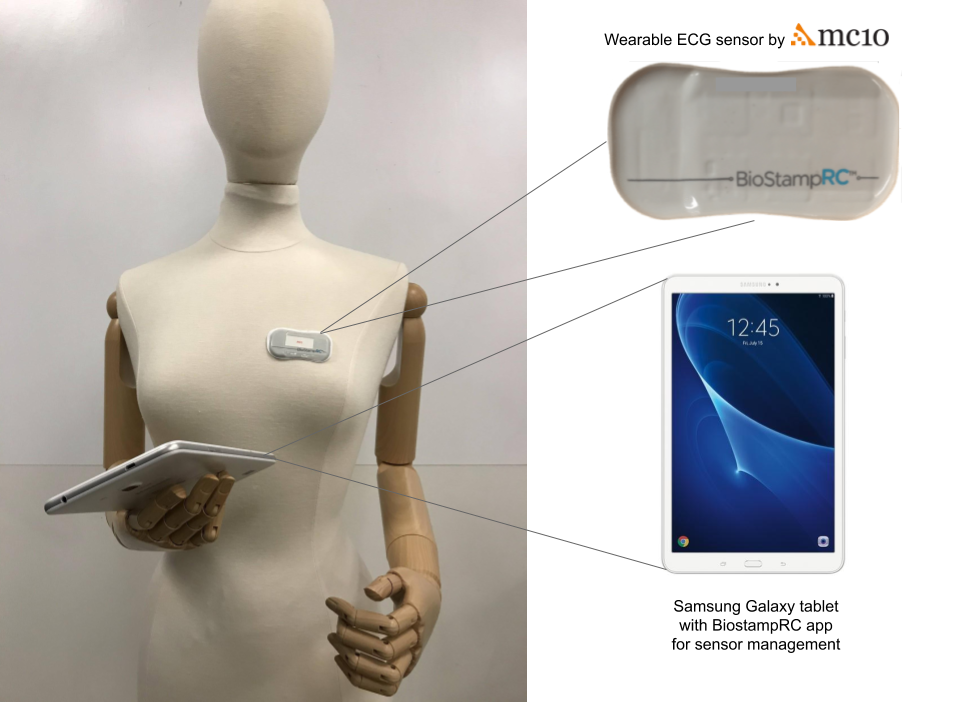


Supplementary Table 1. EMA questionnaire.

| Question No. | Parameter | Emotion | Question | Possible Responses^a^ |
| --- | --- | --- | --- | --- |
| 1 | Binary stress | ‒ | In the past hour, did you experience anything stressful? | Yes/no |
| 2 | Likert stress | ‒ | Over the past hour, how stressed were you feeling? | 0‒6 |
| 3 | PSS-Control | ‒ | Over the past hour, did you feel you could not control important things? | 0‒4 |
| 4 | PSS-Overcome | ‒ | Over the past hour, did you feel difficulties piling up so you cannot overcome them? | 0‒4 |
| 5 | Worried | ‒ | Over the past hour, how worried were you feeling? | 0‒100 |
| 6 | Sad | ‒ | Over the past hour, how sad were you feeling? | 0‒100 |
| 7 | Angry | ‒ | Over the past hour, how irritable/angry were you feeling? | 0‒100 |
| 8 | PSS-Confident | + | Over the past hour, did you feel confident in your ability to handle problems? | 0‒4 |
| 9 | PSS-YourWay | + | Over the past hour, did you feel things are going your way? | 0‒4 |
| 10 | Content | + | Over the past hour, how content were you feeling? | 0‒100 |
| 11 | Happy | + | Over the past hour, how happy were you feeling? | 0‒100 |
| 12 | Excited | + | Over the past hour, how excited were you feeling? | 0‒100 |

+, positive emotion; ‒, negative emotion; EMA, ecological monetary assessment; PSS, perceived stress scale.

^a^For questions 3 and 4: 0 = not at all; 1 = a little bit; 2 = somewhat; 3 = very much; and 4 = extremely. For questions 8 and 9: 0 = extremely; 1 = very much; 2 = somewhat; 3 = somewhat; and 4 = not at all.

Supplementary Table 2. Extracted features used in the model.

| Extracted Feature | Python Name | Extracted Feature |
| --- | --- | --- |
| Intervention based | Intervention_Day | Intervention day |
|  | Count_Intervention | Count intervention |
|  | JIT_Intervention_Day | JIT intervention day |
|  | Count_JIT_Intervention | Count JIT intervention |
| Heart rate variability based | mean, median, mode | Mean, median, mode |
|  | Count, count<mean, count>mean | count, count$>$mean, count$<$mean |
|  | standard deviation | standard deviation |
|  | COV_M | covariance |
|  | min, max, range | Minimum, maximum, range |
|  | RMS | root mean square (RMS) |
|  | Kurtosis | kurtosis |
|  | Skew | skew |
|  | IQR | IQR |
|  | zcross | zero cross |
|  | 20_percentile, 40_percentile, 60_percentile, 80_percentile | 20^th^, 40^th^, 60^th^, 80^th^ percentile |
|  | RMSSD, SDSD | RMSSD, SDSD |
|  | pNN20, pNN50, nn20, nn50 | pNN20, pNN50, nn20, nn50 |
|  | LF, MF, HF, LF/HF | Low-frequency energy (0.1-0.2 Hz), medium-frequency energy (0.2-0.3 Hz), high-frequency energy (0.3-0.4 Hz), Low-:high-frequency energy ratio |
| Duration-based | Total_Stress | Total number of stress-positive minutes in the day |
|  | Total_Consecutive_Stress, Total_Consecutive_Stress_2min, Total_Consecutive_Stress_5min,  Total_Consecutive_Stress_10min | Total number of consecutive stress-positive minutes at 1-, 2-, 5-, and 10-minute thresholds |
|  | Num_Consecutive_Stress, Num_Consecutive_Stress_2min, Num_Consecutive_Stress_5min,  Num_Consecutive_Stress_10min | Number of consecutive stress-positive episodes at 1-, 2-, 5-, and 10-minute thresholds |
|  | Total_Consecutive_Stress_Perc, Total_Consecutive_Stress_Perc_2min, Total_Consecutive_Stress_Perc_5min,  Total_Consecutive_Stress_ Perc _10min | Percentage of consecutive stress-positive minutes at 1-, 2-, 5-, and 10-minute thresholds (Number of consecutive stress-positive minutes over total number of stress-positive minutes) |
|  | Num_Consecutive_Stress_Perc, Num_Consecutive_Stress_Perc_2min, Num_Consecutive_Stress_Perc_5min,  Num_Consecutive_Stress_ Perc _10min | Percentage of consecutive stress-positive episodes at 1-, 2-, 5-, and 10-minute thresholds (Number of episodes of consecutive-stress over total number of stress-positive episodes) |
| Covariates | Age | Age |
|  | Gestation_week | Gestational age at enrollment |
|  | no_of_pregnancies | Number of prior pregnancies |
|  | children | Number of prior children |
|  | depression_score | EPDS score at enrollment |
| EMA-based | PSS4 | PSSQ-4 |
|  | BinaryStress | Binary stress |
|  | LikertStress | Likert stress |
|  | PSSControl | PSS-Control |
|  | PSSOvercome | PSS-Overcome |
|  | WorriedStress | Worried |
|  | SadStress | Sad |
|  | AngryStress | Angry |
|  | PSSConfident | PSS-Confident |
|  | PSSYourWay | PSS-YourWay |
|  | ContentStress | Content |
|  | HappyStress | Happy |
|  | ExcitedStress | Excited |

Supplementary Table 3. Participant characteristics. Participants 8 and 11’s data were excluded from analysis due to low adherence.

| **ID** | **Age** | **Gestational Age at Enrollment** | **No. of Prior Children** | **No. of Prior Pregnancies** | **EPDS Score (Depression Score)** |
| --- | --- | --- | --- | --- | --- |
| 1 | 36 | 11 | 1 | 3 | 10 |
| 2 | 31 | 11 | 0 | 1 | 8 |
| 3 | 36 | 17 | 0 | 1 | 1 |
| 4 | 35 | 13 | 1 | 2 | 5 |
| 5 | 39 | 13 | 2 | 5 | 13 |
| 6 | 34 | 11 | 1 | 3 | 9 |
| 7 | 36 | 12 | 1 | 2 | 5 |
| 9 | 35 | 17 | 0 | 1 | 6 |
| 10 | 37 | 16 | 2 | 3 | 5 |
| 12 | 34 | 14 | 1 | 3 | 10 |
| 13 | NA | 11 | 0 | 1 | 9 |
| 14 | 30 | 10 | 0 | 1 | 3 |
| 15 | 36 | 11 | 2 | 3 | 11 |
| 16 | 34 | 10 | 1 | 2 | 5 |
| **Mean (SD)** | 34.9 (2.4) | 12.6 (2.5) | 0.8 (0.8) | 2.2 (1.2) | 7.2 (3.4) |

Supplementary Table 4. Sensor data captured across 13 of the 16 enrolled participants. Data from three participants were outliers in wear time and excluded from analysis.

| **ID** | **Days Worn** | **Consecutive Days Worn** | **Wear Time (Hours)** | **Clean Data (%)** | **Mean (SD) Wear Hours per Day** |
| --- | --- | --- | --- | --- | --- |
| 1 | 34 | 24 | 455.78 | 92.45 | 13.41 (2.95) |
| 2 | 58 | 42 | 797.93 | 94.95 | 13.76 (2.42) |
| 3 | 23 | 9 | 211.87 | 86.96 | 9.21 (4.17) |
| 4 | 40 | 34 | 421.06 | 75.55 | 10.53 (4.4) |
| 5 | 33 | 27 | 427.72 | 79.47 | 12.96 (3.88) |
| 6 | 5 | 4 | 65.63 | 87.9 | 13.13 (4.8) |
| 7 | 25 | 19 | 324.54 | 95.33 | 12.98 (4.24) |
| 9 | 9 | 7 | 106.36 | 72.04 | 11.82 (3.82) |
| 10 | 5 | 4 | 70.86 | 94.27 | 14.17 (4.22) |
| 12 | 6 | 3 | 87.21 | 96.46 | 14.53 (1.38) |
| 13 | 22 | 16 | 245.55 | 82.71 | 11.16 (3.45) |
| 14 | 68 | 59 | 787.95 | 98.93 | 11.59 (2.27) |
| 16 | 13 | 8 | 130.1 | 64.9 | 10.01 (3.87) |
| **Total** | 344 | 256 | 4157.18 | 89.15 |  |
| **Mean (SD)** | 21.5 (20.7) | 16 (17.2) | 259.8 (258.4) | 88.2 (10.6) | 11.5 (2.8) |

Supplementary Table 5. EMA data captured for 14 of the 16 enrolled participants. Data from two participants were outliers in number of responses and excluded from analysis.

| **ID** | **Days Answered** | **Consecutive Days Answered** | **Total EMAs Answered** | **Mean (SD) EMAs Answered per Day** |
| --- | --- | --- | --- | --- |
| 1 | 83 | 77 | 338 | 4.07 (1.08) |
| 2 | 86 | 81 | 277 | 3.55 (1.48) |
| 3 | 72 | 66 | 115 | 1.62 (0.68) |
| 4 | 84 | 83 | 302 | 3.68 (1.0) |
| 5 | 68 | 54 | 77 | 1.24 (0.53) |
| 6 | 68 | 65 | 211 | 3.1 (1.36) |
| 7 | 61 | 54 | 169 | 2.77 (1.31) |
| 9 | 76 | 75 | 346 | 4.55 (0.93) |
| 10 | 75 | 74 | 304 | 4.05 (1.13) |
| 12 | 43 | 31 | 64 | 1.49 (0.63) |
| 13 | 30 | 24 | 75 | 2.5 (1.14) |
| 14 | 94 | 93 | 340 | 4.53 (0.6) |
| 15 | 44 | 39 | 106 | 2.41 (1.19) |
| 16 | 70 | 65 | 114 | 1.65 (0.7) |
| **Total** | 956 | 881 | 2838 |  |
| **Mean (SD)** | 68.3 (17.9) | 62.9 (20.4) | 202.7 (111.2) | 2.9 (1.2) |

Supplementary Table 6. Hyperparameters of random forest model after Bayesian optimization.

| Hyperparameter | Value After Optimization |
| --- | --- |
| n_estimators | 15 |
| criterion | ‘entropy’ |
| max_depth | 3 |
| min_samples_split | 2 |
| max_features | ‘sqrt’ |

Supplementary Table 7. Predicting next-day physiologic stress by six different machine learning models using 5-fold cross-validation.

| Classifier | Precision (mean) | Recall (mean) | F1 (mean) |
| --- | --- | --- | --- |
| GBM | 0.754 | 0.825 | 0.787 |
| SVM (rbf-kernel) | 0.745 | 0.963 | 0.832 |
| Decision Tree | 0.729 | 0.755 | 0.739 |
| Naïve Bayes | 0.762 | 0.913 | 0.827 |
| AdaBoost | 0.764 | 0.837 | 0.796 |
| Random Forest | 0.795 | 0.886 | 0.836 |

Supplementary Table 8. Predicting next-day perceived stress by six different machine learning models using 5-fold cross-validation.

| Classifier | Precision (mean) | Recall (mean) | F1 (mean) |
| --- | --- | --- | --- |
| GBM | 0.727 | 0.773 | 0.733 |
| SVM (rbf-kernel) | 0.694 | 0.595 | 0.612 |
| Decision Tree | 0.633 | 0.678 | 0.651 |
| Naïve Bayes | 0.688 | 0.573 | 0.596 |
| AdaBoost | 0.662 | 0.721 | 0.653 |
| Random Forest | 0.730 | 0.775 | 0.744 |
